# Supplementary material for: Development and validation of small animal models for onchocerciasis and loiasis microfilaricide discovery
Source: PLoS Negl Trop Dis. 2023 Feb 24;17(2):e0011135. doi: 10.1371/journal.pntd.0011135 (PMC9994675; doi:10.1371/journal.pntd.0011135)
Supplement: Attachment — Submitted filename: Responses to Reviewers Comments (PNTD-D-2-00617).docx [file pntd.0011135.s001.docx]

UNIVERSITY OF BUEA	**REPUBLIC OF	CAMEROON**

**PEACE – WORK – FATHERLAND**

P.O. Box 63

Buea, South West Region

Fax: (237) 33 32 22 72

CAMEROON

Tel: (237) 33 32 21 34 / 33 32 26 90/33 32 27 06

Email: fs.ub@ubuea.cm

**FACULTY OF SCIENCE**

Dean: **Prof. Roland N. NDIP**

Vice-Dean/PAA: **Dr. Jane-Francis Akoachere**

Vice-Dean SSA: **Prof. Achidi Eric Akum**

Vice-Dean RC: **Dr. Achianga Patrick Njukeng**

Faculty Officer: **Mr Emmanuel Mosinge**	Ref No: 2022/_______/UB/FS/BMB

Date: 5^th^ October, 2022

Dear Section Editor,

We are very grateful for the editorial process and the feedback received from the reviewers which has helped to improve the quality of our manuscript.

Please find below the responses provided to reviewers’ comments and the description of the changes made in the manuscript.

***********************

Reviewer's Responses to Questions

<b>Key Review Criteria Required for Acceptance?</b></br></br>

As you describe the new analyses required for acceptance, please consider the following:</br></br>

<b>Methods</b></br></br>

-Are the objectives of the study clearly articulated with a clear testable hypothesis stated?</br>

-Is the study design appropriate to address the stated objectives?</br>

-Is the population clearly described and appropriate for the hypothesis being tested?</br>

-Is the sample size sufficient to ensure adequate power to address the hypothesis being tested?</br>

-Were correct statistical analysis used to support conclusions?</br>

-Are there concerns about ethical or regulatory requirements being met?</br></br>

Reviewer #1: The study objectives are clearly stated, and the study design is acceptable given the constraints the Loa loa and Onchocerca ochengi models come with. It is great to see some exploration of O. ochengi and L. loa mf location in infected gerbils (Table 1) and mice (Table 2). Sample size and statistical analysis are appropriate. No ethical or regulatory requirements concerns.

Reviewer #2: The objectives and design of the study are clearly stated and there are no concerns with regard to the sample size, statistical analysis or ethics. However, the material &methods section needs in parts some more information:

- Please indicate that you used female and male rodents.

*It has been included that “*Similar numbers of both males and female animals were used.” (lines 140-141)

- Indicate the needle gauge size that was used to inject mf.

*It has been included that “*using a 27G needle syringe” (lines 173-174)

- Specify the vehicle used for oral ivermectin treatment (1% DMSO in aqua dest?).

*The 1% DMSO was in PBS. It has been included that “*in PBS” (lines 197, 258, 301, 316, 334)

- Please indicate how you calculated the frequency motility of the microfilariae shown in Fig. 4 (how does a motility of 50% or 75% look like).

*The frequency (proportion) of mf at each motility score was obtained by dividing the number of mf at that motility score by the total number of mf in the volume analyzed (500 µl) and multiplying by 100. To clarify on this, lines 225 following has been modified to read ”* The number of *L. loa* mf in 500 µl was counted and each scored for motility using an inverted microscope. Motility scores of 0% (complete inhibition of motility), 25% (only head or tail moving or vibrating), 50% (worm sluggish), 75% (little change in motility), or 100% (no observable inhibition of motility) were assigned to mf recovered from each animal. The proportion of mf at each motility score was obtained by dividing the number of mf at that motility score by the total number of mf and multiplying by 100. (lines 229-230)

One major limitation of the L. loa jird study is that mf were not intravenously injected. Injection of L. loa mf intravenously in jirds using the vena saphena should be doable without anesthesia (in comparison to the jugular vein). This would allow a direct comparison to the i.v. injection of mice and immobile L. loa mf are likely to be cleared from the jird peripheral blood, which better resembles the human host. Thus, you should repeat the L. loa jird experiment with ivermectin treatment after i.v. injection of L. loa mf, which likely results in a better clearance of mf.

*Thank you for highlighting this major limitation of our study which we clearly made mention of in the manuscript and are looking forward to addressing it. The last two times we went to the field to collect L. loa mf blood from our donors, we noticed that their mf load has dropped drastically. The main challenge we have at the moment is that we need to find new L. loa mf blood donors. The donors we worked with had mf load 15,000 mf/ml and above, but from last samples collected, the load had dropped to <4,000 mf/ml. With this load, we need at least 10 ml of blood to isolate 40,000 mf (number for iv administration) for one gerbil. So for 12 animals for a complete experiment, we need at least 120 ml of blood. Our ethical permit allows us to collect 20 ml of blood per donor each time. We were working with 3 donors, so collecting from 3 of them at once will not be enough. We plan to screen more people to find new consenting donors with high mf loads from whom we can be collecting blood. This involves mobilization of the villagers through community leaders they trust and sensitization them on the importance of the study. They are usually very skeptical, so even after screening, you will need to convince them to be blood donors for the research. Our laboratory is located 150 km from the area where we plan to do this new screen.*

*While we will like to do this experiment immediately, the logistics involved requires a lot more time to get new L. loa mf blood donors. Regrettably, we will not be able to do L. loa mf intravenous administration in gerbils to include in this manuscript for the above-stated reason. Nevertheless, we showed that ivermectin can lead to a reduction of L. loa mf in gerbils (albeit the peritoneum) and that given the limitations, gerbils can be still be used as an animal model in addition to the mouse models.*

For the co-infection experiment (please call it a mf co-injection experiment), human whole blood containing L. loa mf were i.p. injected in gerbils (line 180). The injection of human whole blood likely induces immune responses that affect the response to mf as well. Why did the authors chose this method? Does the injection of purified L. loa mf followed by O. ochengi mf result in a similar outcome?

*Co-infection has been replaced by co-injection in 14 different places (lines 41, 43, 62, 184, 185, 196, 319, 330, 338, 356, 372, 438, 444, 463)*

*We injected human whole blood for two reasons: firstly, we thought it will help the mf to be more viable in the gerbils and enter the bloodstream since they will still be in human blood (their natural environment) and not go through the lengthy purification process which could result in a faster drop in viability. However, this did not help the L. loa mf to enter the bloodstream of the gerbils. Secondly, when the L. loa mf load was not very high (>30,000/ml), we preferred to inject the whole blood because some mf are lost during the purification process.*

*It is very likely that injection of human whole blood may induce immune responses giving that blood has diverse component. However, the results obtained from injecting purified L. loa mf (Fig. 4) and mf in whole human blood (Fig. 5) follow the same trend.*

The different ivermectin treatment regimens and concentrations used do not allow a direct comparison of the models to assess the susceptibility of the microfilariae of both filarial species to ivermectin. This is a major limitation of the study. The authors should at least discuss this limitation of their study and compare theirs doses used with previously published concentrations of ivermectin in rodent models.

*The human dose of ivermectin is 150 µg/kg through the oral route. We used this dose and route for both mice and gerbils and it cleared all O. ochengi mf in the earlobes (Fig. 1). In our previous study[1], we equally showed that this same dose eliminates all O. ochengi mf in hamsters. Thus the dose of 150 µg/kg is very effective against O. ochengi mf in different animal models.*

*However, the same dose of 150 µg/kg non-significantly decreased the number of L. loa mf in the peritoneum and reduced viability (Fig. 4) in treated gerbils compared to control gerbils. As expected, all O. ochengi mf were eliminated. To improve on the effect of IVM, the dose was increased to 1mg/kg but this still resulted in a non-significant decrease in L. loa mf in the peritoneum of treated animals compared to controls (Fig. 5). However, the percentage reduction in L. loa mf in the peritoneum moved from 34.24 to 55.44 with 150 µg/kg and 1mg/kg (two doses of 0.5 mg/kg body weight) IVM respectively. A similar dose of 1mg/kg IVM given to mice, resulted in a significant reduction in the number of L. loa mf in the lungs and in the blood (Fig. 3). This indicates that the difference in the effect of IVM lies in the compartment where the mf are found and could also be due to bioavailability of the drug. IVM (1mg/kg) resulted in 81.63 %* *reduction in L. loa mf in cardiac blood which is similar ~92% reduction obtained in a CB.17 SCID mice model[2].*

*This discussion has been modified as follows (lines 423 – 437) “The human dose of IVM is 150 µg/kg body weight through the oral route. Using this dose and route for both mice and gerbils resulted in complete elimination of O. ochengi mf in the earlobes (Fig. 1), similar to our previous observation in hamsters [22]. However, this same dose led to decrease in mf motility but non-significant reduction of number of mf in the peritoneum (Fig. 4).* Increasing the dose of IVM from a single dose of 150 µg/kg to two doses of 0.5 mg/kg body weight led to a reduction of *L. loa* mf in the peritoneum by 34.24% and 55.44%, respectively. This suggests that increasing the dose of IVM could lead to a significant decrease in *L. loa* mf in the peritoneum. *A similar dose of 1mg/kg IVM given to mice, resulted in a significant reduction in the number of L. loa mf in the lungs and in the blood (Fig. 3), akin to the results obtained with 10 mg/kg. This indicates that the difference in the effect of IVM may lie in the compartment where the mf are found and could also be due to bioavailability of the drug. IVM (1mg/kg) resulted in 81.63 % reduction in L. loa mf in cardiac blood which is similar to ~92% reduction obtained in a CB.17 SCID mice model [29].“*

--------------------

<b>Results</b></br></br>

-Does the analysis presented match the analysis plan?</br>

-Are the results clearly and completely presented?</br>

-Are the figures (Tables, Images) of sufficient quality for clarity?</br></br>

Reviewer #1: The results are clear and coherent; however I would suggest to add a little more information on the graphs themselves as it gets confusing. For instance, for Figure 1 it would be good to mention in the y axis that in panel A this is in gerbils and in panel B in mice. This would avoid to always refer to the legend to remember which one is which. Same suggestion for Figure 6.

*Fig.1. Gerbil and mice have been clearly indicated on the y-axis of panel a and b respectively*

*Fig.6. 40 days pi and 50 days pi have been added to the y-axis label of panel a and b respectively*

Reviewer #2: The data is clearly presented. Please indicate the gender of the animals used in all figure legends.

*In all experiments, we used both male and female animals (lines 140-141). The gender of the animals was matched in both the control and test groups. The gender of the animals used has been indicated on all the figure legends (lines 259-260, 285-286, 298-299, 313-314, 330, 358)*

Table 2 indicates that you have indeed on average the highest L. loa mf counts in the lung tissue, but only 5 out of 9 mice had mf in the lung tissue at day 8 after mf injection. In contrast, 8 of 9 mice had still mf present in the heart tissue at that time point. So in order to confirm presence of mf at later time points in mice, the heart tissue may be a better indicator. Do you have data from the mf burden from the heart tissue at 5 and 6 days after mf injection (Fig. 2A, B)? This should be added to the results section. Additional analyses of L. loa mf from the heart tissue of mice may allow the assessment of drugs that lead to a slower L. loa mf removal as ivermectin in mice. This should be discussed.

*We thank the reviewer for this keen observation. After the 8 days experiments, we decided to focus on checking L. loa mf in cardiac blood and in the tissue where the mf were most abundant which is the lungs. Therefore we do not have mf data for heart tissue on day 5 and 6. Although many more mice had L. loa mf in the heart tissue on day 8, the low number of mf recovered downplays it as the best tissue for further analysis at least at this time point.*

*The discussion has been modified in lines 404 – 408 to read “*By day 8, the percentage recovery was 0.08, 0.09 and 0.66 % in cardiac blood, heart and lung tissues respectively, indicating ~8 times more likelihood to find *L. loa* mf in the lung tissue than in cardiac blood and tissue. The lung tissue is therefore a valuable site for the analysis of the effect of slow acting drugs on *L. loa* mf in BALB/c mice.”

--------------------

<b>Conclusions</b></br></br>

-Are the conclusions supported by the data presented?</br>

-Are the limitations of analysis clearly described?</br>

-Do the authors discuss how these data can be helpful to advance our understanding of the topic under study?</br>

-Is public health relevance addressed?</br></br>

Reviewer #1: Four main points of concern in this section:

1)    After 30 days infection, only 1% O. ochengi mf (232.5 mf recovered on average for 22725 mf inoculated, Table 1) were recovered in ear lobes compared to 5% after 14 days – why has D14 not been chosen over D30 for IVM challenge studies?

*Day 30 was chosen because we wanted to leave enough window that can permit us see if the model could be used to assess the effect of slow acting drugs. This is the reason we further assessed if the mf could survive for up to 50 days in the gerbils. Considering that the phenotype with IVM is a black and white one (IVM eliminates all O. ochengi mf), it really did not matter whether this effect is assessed on day 14 or day 30.*

2)    Due to the rapid elimination of Loa mf in immunocompetent animals (almost no mf left in the lungs after 8 days, Figure 2), a very short therapeutic window is therefore left for testing drugs for their effects on L. loa mf. It would be interesting to investigate whether the number of injected mf can be increased so blood circulating microfilaremia could be the parameter of choice when it comes to evaluate mf killing. Mf localised in the lungs veins could just be trapped there and therefore not reflect true circulating and mobile mf.

*We thank the reviewer for this very good suggestion. This will be considered in our future experiments when we can get enough L. loa mf to do a dose dependent analysis. The major limitation here is to find people that have extremely high loads of L. loa mf in the blood that can permit isolation of enough mf for the experiment.*

3)    As outlined by authors, the co-infection model still needs to be optimized: mode of infection (IV injection to be validated for Loa mf), mf loads (it might be judicious to increase mf loads), IVM dose and injection site (perhaps high dose IVM 5mg/kg subcut as in ref 29), readout time point (might need to be brought forward, especially in the context of co-infections).

*Indeed as we mentioned, the model needs optimization. Increasing mf load has been quite challenging as it depends of L. loa mf loads of donors which usually fluctuates. High dose of IVM (5mg/kg administered subcutaneously as used in [2]) could result in significant reduction in L. loa mf in gerbils. Once we get new donors with high L. loa mf loads, all these will be considered in our optimization experiments.*

4)    Have authors tested other anti-filarial drugs? It would be good to confirm that macrofilaricidal benzimidazole drugs such as flubendazole or oxfendazole don’t have any off-target effect on L. loa mf loads.

*Yes we tested other anti-filarial drugs such as pyrvinium pamoate (PVP) and tetrahydropyrvinium (THP) [3]. From the results obtained we could confirm that there was no off-target effect on L. loa mf in mice. This will be published in another article.*

***Anti-filarial drugs have no effect on L. loa mf in BALB/c mice.*** *Mice were infected intravenously with 40,000 L. loa mf. After 12 hours, mice were orally treated with 1 mg/kg (mpk) body weight IVM or intravenously with PVP, THP and ARTA-022 at 0.5 mg/kg body weight SID. Control animals received vehicle (1% DMSO in PBS). Mice were analyzed 2.5 days post-infection for L. loa mf in (A) Lungs, and (B) Cardiac blood. *P<0.05*

*Similarly, it was shown that macrofilaricidal benzimidazole drugs such as flubendazole or oxfendazole did not target L. loa mf in mice unlike IVM (Pionnier et al., Nature Comm, 2019).*

Reviewer #2: There is a need for new animal models to test drug candidates for filarial diseases. Especially a better understanding of drug efficacy against microfilariae of L. loa and O. volvulus is important, as indicator for the safety profile in future clinical trials. Thus, this study is of relevance to the field. This aspect is clearly presented in the discussion. However, limitations of the study, such as the different ivermectin doses and regimens have to be mentioned. Furthermore, the authors should highlight why it is important to have immunocompetent animal models to assess drug efficacy. There are several publications that indicate that efficacy of drugs such as benzimidazoles, ivermectin and DEC are supported by the immune system.

*The dose of IVM used in both mice and gerbils against O. ochengi mf is a single dose of 150 µg/kg body weight which is the same dose given to humans. This dose was not effective against L. loa mf (Fig. 4), thus we used 1 and 10 mg/kg in mice and two doses of 0.5* *mg/kg IVM was administered 8 hours apart in gerbils. One (1) mg/kg oral and 5 mg/kg subcutaneous dose of IVM has been shown to significantly reduce L. loa mf in mice (Pionnier et al., Nature Comm, 2019).*

*This discussion has been modified as follows (lines 423 – 437) “The human dose of IVM is 150 µg/kg body weight through the oral route. Using this dose and route for both mice and gerbils resulted in complete elimination of O. ochengi mf in the earlobes (Fig. 1), similar to our previous observation in hamsters [22]. However, this same dose led to decrease in mf motility but non-significant reduction of number of mf in the peritoneum (Fig. 4).* Increasing the dose of IVM from a single dose of 150 µg/kg to two doses of 0.5 mg/kg body weight led to a reduction of *L. loa* mf in the peritoneum by 34.24% and 55.44%, respectively. This suggests that increasing the dose of IVM could lead to a significant decrease in *L. loa* mf in the peritoneum. *A similar dose of 1mg/kg IVM given to mice, resulted in a significant reduction in the number of L. loa mf in the lungs and in the blood (Fig. 3), akin to the results obtained with 10 mg/kg. This indicates that the difference in the effect of IVM may lie in the compartment where the mf are found and could also be due to bioavailability of the drug. IVM (1mg/kg) resulted in 81.63 % reduction in L. loa mf in cardiac blood which is similar to ~92% reduction obtained in a CB.17 SCID mice model [29].“*

*Since our study did not look at any immunological aspect, we will like avoid delving into immunological effect of anti-filarial drugs. Besides, some publications show/suggest that benzimidazole like flubendazole, IVM and sometimes DEC have a direct effect on the parasite (Wilson et al., Parasite & Vectors, 2021; Lacey, Int J Parasito., 1988; Verma et al., Communications Biology, 2020).*

*This sentence has been added ”* *While some drugs act directly on the parasite, others exploit the immune system to be effective*.” (lines 367-368) *in the following context*

There are a very limited number of animal models available to study onchocerciasis and loiasis. These models are mainly non-human primates or immuno-deficient rodents which are limiting for robust exploration of these diseases. *While some drugs act directly on the parasite, others exploit the immune system to be effective.* This study was therefore aimed at developing immunocompetent small laboratory animal models to study these diseases with a prime focus on the microfilariae. (lines	365-370)

--------------------

<b>Editorial and Data Presentation Modifications?</b></br><br/>

Use  this section for editorial suggestions as well as relatively minor modifications of existing data that would enhance clarity. If the only modifications needed are minor and/or editorial, you may wish to recommend “Minor Revision” or “Accept”.

Reviewer #1: Minor revisions needed – see conclusions section for more details regarding requests

Reviewer #2: The first two sentences of the abstract and the author summary are exactly the same. The author summary should be rewritten, so that it does not duplicate the abstract but highlights the importance of this study in a broader context.

The first two sentences of the author summary have been changed to *Onchocerciasis (river blindness) caused by the filarial worm Onchocerca volvulus is a severe skin disease and affects the eyes leading to vision loss. Drugs that are effective against O. volvulus should be ineffective against another filarial worm Loa loa, as severe adverse events has been reported in treated co-infected individuals. (lines 49-56)*

Indicate in the introduction (line 109) why the use of immunocompromised hosts is a potential issue for preclinical research.

*The following sentence has been added “Some drugs act through the immune system, so it is preferable not to use immunocompromised animals in preclinical studies.” (Lines 113-114)*

In general, injection of microfilariae should not be called an infection. Please change this accordingly throughout the manuscript. E.g. line 106 “7 days post-infection” to “7 days post mf injection” to clarify that no adult worm infection was present. Line 167, 172 etc.

*Infection has been changed to injection throughout the manuscript (lines 41, 43, 62, 182, 184, 185, 196, 250, 319, 330, 338, 356, 360, 372, 421, 438, 444, 463)*

Line 374: change to “…validating both HOSTS for microfilariae studies”.

*“Models” have been replace by “hosts” (lines 387)*

--------------------

<b>Summary and General Comments</b></br></br>

Use  this section to provide overall comments, discuss strengths/weaknesses of the study, novelty, significance, general execution and scholarship. You may also include additional comments for the author, including concerns about dual publication, research ethics, or publication ethics. If requesting major revision, please articulate the new experiments that are needed.

Reviewer #1: Developing in vivo drug screening models for filariasis has been (and still is) a challenge. There are several models available right now but many if not all need to rely on modifications brought to the host immune system (immunodeficiencies, splenectomies, specific depletion treatment) or to the use of surrogate parasite species. The authors have therefore concentrated their efforts in using immunocompetent animals in the context of Loa and Onchocerca infections. Despite some limitations (use of O. ochengi, IP injections instead of IV for the gerbil model), they obtained interesting results (especially in the context of IVM treatment) which could lead to some potential for their models to be used for drug screening. However, some aspects would still need to be explored in more details and optimized (mf loads, readout time point, readout parameters, co-infection model). It would be good if the authors could address the raised concerns in the conclusions section

of the review before a final decision is made on the manuscript acceptance for publication.

*We thank the reviewer very much for all the feedback provided and we have addressed the issues raised.*

Reviewer #2: The present manuscript describes the potential of immunocompetent BALB/c mice and gerbils that are injected with mf of L. loa or O. ochengi as model to assess the microfilaricidal efficacy of drug candidates. There is a need of such models, as the present small rodent models mainly use immunocompromised mouse strains despite the fact that the immune system may contribute to drug efficacy. One of the major concerns is the intraperitoneal injection of L. loa mf containing whole blood in jirds. The injection of human blood likely induces pro-inflammatory immune response. Furthermore, as seen by the results, intraperitoneally injected mf are not migrating into the peripheral blood and are not cleared by ivermectin treatment. Thus, this experiment should be repeated with i.v. injection of L. loa mf. Another issue is the use of different doses and regimens of ivermectin for the different experiments, which limits the comparisons of the different outcomes. As an

additional control, a drug that is mainly macrofilaricidal, such as oxfendazole, should have been once validated.

*We are very grateful to the reviewer for a thorough review of the manuscript and have provided responses to the issues raised. Regrettably, we cannot repeat the i.v. injection of gerbils at this time because of the logistics involved in getting infected donors as explained in details above. We tested other filaricides such as pyrvinium pamoate (PVP) and tetrahydropyrvinium (THP), and showed that it had no effect on L. loa mf in mice.*

--------------------

PLOS authors have the option to publish the peer review history of their article (<a href="<https://journals.plos.org/plosntds/s/editorial-and-peer-review-process#loc-peer-review-history>" target="_blank">what does this mean?</a>). If published, this will include your full peer review and any attached files.<br><br>

If you choose “no”, your identity will remain anonymous but your review may still be made public.<br><br>

<b>Do you want your identity to be public for this peer review?</b> For information about this choice, including consent withdrawal, please see our <a href="<https://www.plos.org/privacy-policy>" target="_blank">Privacy Policy</a>.

Reviewer #1: No

Reviewer #2: No

Figure Files:

While revising your submission, please upload your figure files to the Preflight Analysis and Conversion Engine (PACE) digital diagnostic tool, <https://pacev2.apexcovantage.com. PACE> helps ensure that figures meet PLOS requirements. To use PACE, you must first register as a user. Then, login and navigate to the UPLOAD tab, where you will find detailed instructions on how to use the tool. If you encounter any issues or have any questions when using PACE, please email us at [figures@plos.org.](mailto:figures@plos.org.)

Data Requirements:

Please note that, as a condition of publication, PLOS' data policy requires that you make available all data used to draw the conclusions outlined in your manuscript. Data must be deposited in an appropriate repository, included within the body of the manuscript, or uploaded as supporting information. This includes all numerical values that were used to generate graphs, histograms etc.. For an example see here: http://www.plosbiology.org/article/info%3Adoi%2F10.1371%2Fjournal.pbio.1001908#s5.

Reproducibility:

To enhance the reproducibility of your results, we recommend that you deposit your laboratory protocols in protocols.io, where a protocol can be assigned its own identifier (DOI) such that it can be cited independently in the future. Additionally, PLOS ONE offers an option to publish peer-reviewed clinical study protocols. Read more information on sharing protocols at <https://plos.org/protocols?utm_medium=editorial-email&utm_source=authorletters&utm_campaign=protocols>

__________________________________________________

In compliance with data protection regulations, you may request that we remove your personal registration details at any time.  (Use the following URL: <https://www.editorialmanager.com/pntd/login.asp?a=r>). Please contact the publication office if you have any questions.

**References**

- Lacey E. 1988. The role of cytoskeletal protein, tubulin, in the mode of action and mechanism of drug resistance to benzimidazoles. International Journal for Parasitology, 18, 885-936.

-Mbah GE, Ayiseh RB, Cho-Ngwa F. Development and validation of an *Onchocerca ochengi* microfilarial hamster model for onchocerciasis drug screens. BMC Infect Dis. 2016; 16(1):404.

-Pionnier NP, Sjoberg H, Chunda VC, Fombad FF, Chounna PW, Njouendou AJ, et al. Mouse models of Loa loa. Nature Communications. 2019; 10(1):1429.

-Verma S, Kashyap SS, Robertson AP, Martin RJ. Diethylcarbamazine activates TRP channels including TRP-2 in filaria, Brugia malayi . Communications Biology. 2020;3(1):398. doi: 10.1038/s42003-020-01128-4

-Wilson, N.E., Reaves, B.J. & Wolstenholme, A.J. Lack of detectable short-term effects of a single dose of ivermectin on the human immune system. *Parasites Vectors* **14**, 304 (2021). https://doi.org/10.1186/s13071-021-04810-6

1.	Mbah GE, Ayiseh RB, Cho-Ngwa F. Development and validation of an *Onchocerca ochengi* microfilarial hamster model for onchocerciasis drug screens. BMC Infect Dis2016;16(1):404.

2.	Pionnier NP, Sjoberg H, Chunda VC, Fombad FF, Chounna PW, Njouendou AJ, et al. Mouse models of Loa loa. Nature Communications2019;10(1):1429.

3.	Gunderson EL, Bryant C, Bulman CA, Fischer C, Luo M, Vogel I, et al. Pyrvinium Pamoate and Structural Analogs Are Early Macrofilaricide Leads. Pharmaceuticals2022;15(2):189.

Once more, we are grateful to the Editor and reviewers.

Thank you for your kind consideration of our responses and the changes effected.

Sincerely yours,

ARB

Dr. Rene B. Ayiseh
