# Supplementary material for: Development and validation of small animal models for onchocerciasis and loiasis microfilaricide discovery
Source: PLoS Negl Trop Dis. 2023 Feb 24;17(2):e0011135. doi: 10.1371/journal.pntd.0011135 (PMC9994675; doi:10.1371/journal.pntd.0011135)
Supplement: Attachment — Submitted filename: Addressing reviewers concern 13_11_22_SL.docx [file pntd.0011135.s002.docx]

<b>Summary and General Comments</b></br></br>

Use  this section to provide overall comments, discuss strengths/weaknesses of the study, novelty, significance, general execution and scholarship. You may also include additional comments for the author, including concerns about dual publication, research ethics, or publication ethics. If requesting major revision, please articulate the new experiments that are needed.

Reviewer #1: Thanks to the authors for having provided some extra information regarding the points I rose. I however still have a couple of requests before any decision regarding manuscript acceptance can be made:

1)    I really appreciated the justification points made for the study limitations in the response to reviewers’ section. I believe some interesting points (the challenges in sourcing more Loa mf, the injection of mf in human blood, the controversy around the analyses of L. loa mf from the heart tissue or the dose of IVM used) would benefit from being acknowledged/discussed in the discussion section of the manuscript. The limitations of the models and the next development steps should be clearly addressed.

2)    With all these study limitations and the experiments not having been repeated to confirm the results, I would therefore be prudent on too definitive statements and would temper some of the paper conclusions or statements. For instance, I would remove the “validation” aspect from the paper (title, main text) and make it clearer that some development/validation is still needed (especially in the discussion). The model validation aspect could perhaps be discussed if the interesting PVP/THP work was included in this paper.

Reviewer #2: The authors adequately addressed my previous concerns in their revised manuscript.

--------------------

Reviewer #1: Thanks to the authors for having provided some extra information regarding the points I rose. I however still have a couple of requests before any decision regarding manuscript acceptance can be made:

1)    I really appreciated the justification points made for the study limitations in the response to reviewers’ section. I believe some interesting points (the challenges in sourcing more Loa mf, the injection of mf in human blood, the controversy around the analyses of L. loa mf from the heart tissue or the dose of IVM used) would benefit from being acknowledged/discussed in the discussion section of the manuscript. The limitations of the models and the next development steps should be clearly addressed.

*We are very grateful to the reviewer for all his comments that have helped to improve the quality of our manuscript.*

*The limitations/challenges and steps towards development of a better model mainly applies to the L. loa mf gerbil model. Some aspects of this have already been addressed in the text. The issue of the dose of IVM has already been addressed (see lines 427-436). We do not plan to continue injecting human blood as we clearly explained in our previous reply, that this was done with the hope of getting the L. loa mf from the peritoneum to the blood and it was unsuccessful.*

*The following paragraph has been added to the discussion to address the remaining issues (lines 446-465).*

*The gerbil model for both O. ochengi and L. loa mf appears more attractive as there is longer sustenance of the mf in these animals compared to mice. However, while the O. ochengi mf are well established in the skin of the gerbils as in humans, the gerbil L. loa mf peritoneal model falls short in comparison to the natural blood dwelling mf in humans. Therefore, this model needs to be further developed and validated. The main challenge with the development of this model is having a sustained source of human blood with high loads of L. loa mf. It is very important to have donors with loads of ≥15,000 mf/ml to be able to isolate enough mf to inject, especially when administering 40,000 mf intravenously per animal. We have experienced serious decrease in the mf loads of donors which has considerably advancing our studies further further. In the future we will make sure that we recruit a larger repertoire of donors, so one can easily switch donors if their mf load drops. An alternate source of L. loa mf is the baboon model where mf load can be sustained by injecting the infective L3 stage into the animal [1]. Future experiments aimed to better develop this model will focus on intravenous injection of L. loa mf isolated from human blood followed by validation with orally administered IVM at 150 µg/kg body weight. If this IVM dose does not result in a significant decrease in L. loa mf load, 1 and 10 mg/kg body weight could be considered as was done in the mice model. Furthermore, mf analysis will not only be limited to cardiac blood and lung tissue; the heart tissue will also be considered because it was noticed that 8 out of 9 mice had mf in heart tissue on day 8 whereas only 5 of 9 still had mf in the lungs tissue (Table 2).*

2)    With all these study limitations and the experiments not having been repeated to confirm the results, I would therefore be prudent on too definitive statements and would temper some of the paper conclusions or statements. For instance, I would remove the “validation” aspect from the paper (title, main text) and make it clearer that some development/validation is still needed (especially in the discussion). The model validation aspect could perhaps be discussed if the interesting PVP/THP work was included in this paper.

*In this study, we tested 2 microfilariae (O. ochengi and L. loa) in mice (BALB/c) and gerbils. The results obtained are tabulated below*

| ***SN*** | ***Animal model*** | ***Microfilariae*** | ***Validation with ivermectin*** |
| --- | --- | --- | --- |
| *1* | *Mouse* | *O. ochengi* | *Validated* |
| *2.* | *Gerbil* | *O. ochengi* | *Validated* |
| *3.* | *Mouse* | *L. loa* | *Validated* |
| *4.* | *Gerbil* | *L. loa* | *Not validated. Insignificant reduction observed* |

*From the above summary, 3 of the 4 models tested were validated with ivermectin and the microfilariae were in similar sites/compartment in the animal as in humans. In the manuscript we do not say we validated the L. loa mf in gerbils as we clearly acknowledged the limitation of the model. With these 3 models that are clearly validated, we believe retaining validation in the title and the text (appropriately) is not unfounded.*

In addition, we made some minor modifications throughout the discussion to improve the quality of the write-up without changing any single sentence completely.

Once more, we really appreciate the reviewer for all the comments/feedback.

***Reference***

1.	Wanji S, Eyong E-E, Tendongfor N, Ngwa C, Esuka E, Kengne-Ouafo A, et al. Parasitological, Hematological and Biochemical Characteristics of a Model of Hyper-microfilariaemic Loiasis (*Loa loa*) in the Baboon (*Papio anubis*). PLoS neglected tropical diseases2015;9(11):e0004202.
